# Visualizing Ligand Binding to a GPCR *In Vivo* Using NanoBRET

**DOI:** 10.1016/j.isci.2018.08.006

**Published:** 2018-08-11

**Authors:** Diana C. Alcobia, Alexandra I. Ziegler, Alexander Kondrashov, Eleonora Comeo, Sarah Mistry, Barrie Kellam, Aeson Chang, Jeanette Woolard, Stephen J. Hill, Erica K. Sloan

**Affiliations:** 1 Division of Physiology, Pharmacology and Neuroscience, School of Life Sciences, University of Nottingham, Nottingham NG7 2UH, UK; 2 Centre of Membrane Proteins and Receptors, University of Birmingham and University of Nottingham, The Midlands, UK; 3 Wolfson Centre for Stem Cells, Tissue Engineering & Modelling (STEM), Centre for Biomolecular Sciences, University of Nottingham, Nottingham NG7 2RD, UK; 4 School of Pharmacy, Division of Biomolecular Science and Medicinal Chemistry, Centre for Biomolecular Sciences, University of Nottingham, Nottingham NG7 2RD, UK; 5 Drug Discovery Biology, Monash Institute of Pharmaceutical Sciences, Monash University, Parkville, VIC 3052, Australia; 6 Cousins Center for Neuroimmunology, Semel Institute for Neuroscience and Human Behavior, Jonsson Comprehensive Cancer Center, and UCLA AIDS Institute, University of California Los Angeles, Los Angeles, CA 90095, USA; 7 Division of Surgical Oncology, Peter MacCallum Cancer Centre, Victorian Comprehensive Cancer Centre, 305 Grattan Street, Melbourne, VIC 3000, Australia

**Keywords:** Biological Sciences Tools, Cancer, Molecular Interaction, Optical Imaging

## Abstract

The therapeutic action of a drug depends on its ability to engage with its molecular target *in vivo*. However, current drug discovery strategies quantify drug levels within organs rather than determining the binding of drugs directly to their specific molecular targets *in vivo*. This is a particular problem for assessing the therapeutic potential of drugs that target malignant tumors where access and binding may be impaired by disrupted vasculature and local hypoxia. Here we have used triple-negative human breast cancer cells expressing β_2_-adrenoceptors tagged with the bioluminescence protein NanoLuc to provide a bioluminescence resonance energy transfer approach to directly quantify ligand binding to a G protein-coupled receptor *in vivo* using a mouse model of breast cancer.

## Highlights

• NanoLuc luciferase was used to localize β_2_-adrenoceptors *in vivo*
• Metastatic breast cancer cells retain expression of β_2_-adrenoceptors
• The binding of fluorescent propranolol to β_2_-adrenoceptors was quantified *in vivo*
• NanoBRET was used to monitor target engagement in a mouse model of breast cancer

## Graphical Abstract

**Figure:**
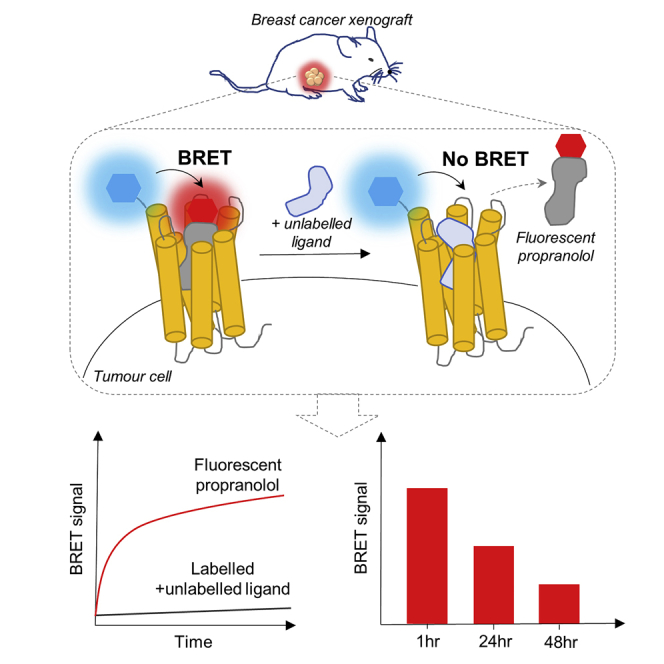

## Introduction

G protein-coupled receptors (GPCRs) comprise the largest family of cell surface receptors involved in signal transduction (Santos et al., 2017, Wacker et al., 2017). In recent years, there has been a realization that a number of GPCRs may play important roles in cancer (Nieto Gutierrez and McDonald, 2018, De Francesco et al., 2017, Bar-Shavit et al., 2016, Liu et al., 2016, O'Hayre et al., 2013). For example, β-adrenoceptors are intimately involved in the pathogenesis of infantile hemangioma (Leaute-Labreze et al., 2008, Stiles et al., 2012) and have been implicated in the progression of several malignant tumor types including angiosarcoma, breast cancer, and ovarian cancer (Rains et al., 2017, Watkins et al., 2015, Armaiz-Pena et al., 2013, Choy et al., 2016, Le et al., 2016, Sloan et al., 2010). In particular, activation of β_2_-adrenoceptors by physiological stress can switch cancer cells to an invasive metastatic phenotype (Sloan et al., 2010, Chang et al., 2016, Creed et al., 2015). The classical β-adrenoceptor antagonist, propranolol, has clinical efficacy for the treatment of infantile hemangiomas and angiosarcomas (Leaute-Labreze et al., 2008, Leaute-Labreze et al., 2015, Chow et al., 2015, Stiles et al., 2013) and prevents the progression of cancer in mouse models (Sloan et al., 2010) and in patients (Shaashua et al., 2017, De Giorgi et al., 2018).

Most cells in the body are close to vasculature allowing easy access of drugs from circulation. However, in tumors, hypoxic regions and tortured non-functional vasculature (Folkman, 1971; Ferrara, 2001, Carmeliet and Jain, 2011, Wong et al., 2015) result in a population of cells that are distant from blood vessels (Carmeliet and Jain, 2011, Minchinton and Tannock, 2006). This distance may hinder the extent to which drugs reach these cells and interact with their molecular target (Minchinton and Tannock, 2006). However, up until now there has been no simple way to visualize directly the extent to which target engagement has been achieved *in vivo*.

One approach to monitor ligand binding to GPCRs in living cells is through the use of fluorescent ligands (Baker et al., 2011, Vernall et al., 2014, Stoddart et al., 2016). However, uptake into cells can often lead to high levels of non-specific binding. We have recently dramatically improved the study of fluorescent ligand binding by developing a bioluminescence resonance energy transfer (BRET) assay that requires close proximity (*circa* 10 nm) between the fluorescent ligand and the target receptor to generate a measurable signal (Stoddart et al., 2015, Stoddart et al., 2018). This has been achieved using GPCRs tagged with a very bright luciferase (NanoLuc; Stoddart et al., 2015, Stoddart et al., 2018). This technological advance allowed us to monitor binding to the human β_2_-adrenoceptor in real time using a red fluorescent analog of the antagonist propranolol, propranolol-(β-Ala-β-Ala)-X-BODIPY 630/650 (Prop-BY630; Stoddart et al., 2015; Figure S1). Here we have used this fluorescent ligand in conjunction with a triple-negative human breast cell line (MDA-MB-231^HM^) expressing an N-terminal NanoLuc-tagged human β_2_-adrenoceptor to quantify ligand binding (using NanoBRET) to a GPCR *in vivo* using a mouse model of breast cancer.

## Results and Discussion

**Figure 1:**
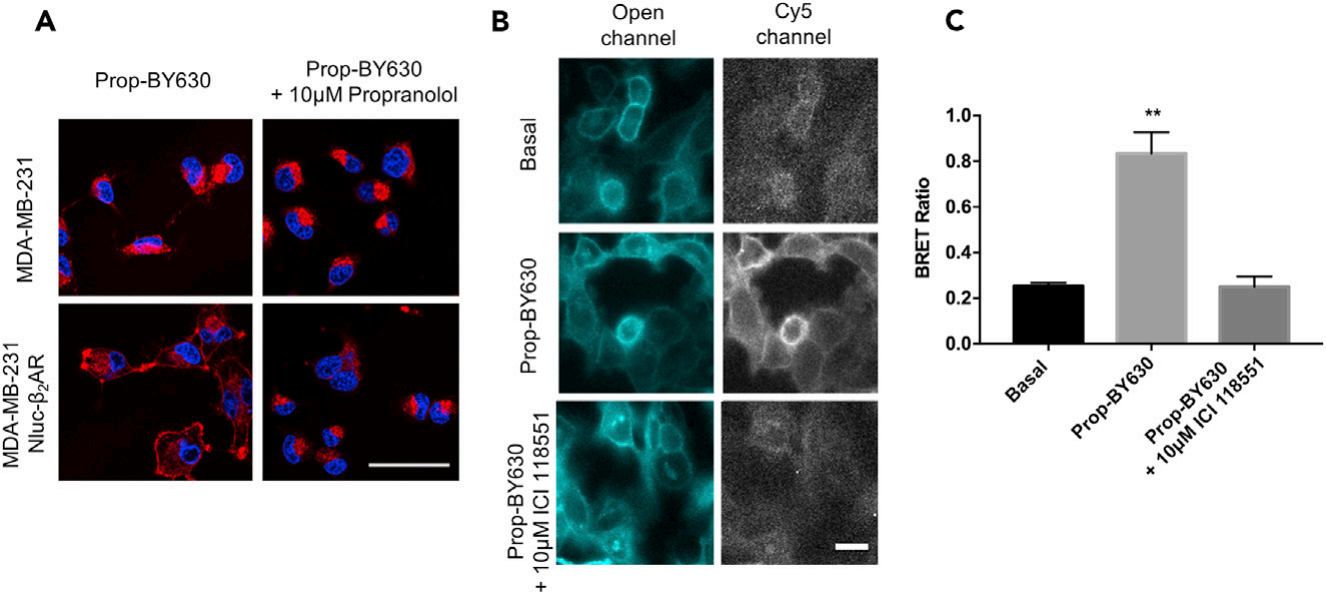
Binding of Fluorescent Propranolol (Propranolol-(β-Ala-β-Ala)-X-BODIPY630/650; Prop-BY630) to Triple-Negative Human Breast Cancer Cells (MDA-MB-231^HM^ cells) (A) Fluorescence imaging of the binding of 50 nM Prop-BY630 to non-transfected MDA-MB-231^HM^ cells (upper panels) or MDA-MB-231^HM^ cells expressing NanoLuc-tagged human β_2_-adrenoceptors (lower panels). Cells were pre-treated with Hoechst 33342 nuclear stain (2 μg/mL; blue labeling) and then labeled for 30 min with Prop-BY630 (red labeling). Upper right and bottom right panels show cells pre-incubated with 10 μM unlabelled propranolol before labeling with fluorescent propranolol (50 nM Prop-BY630). Cells were washed just before imaging to remove unbound fluorescent ligand. Data are representative images from 3 independent experiments. Scale bar represents 50μm. (B) Bioluminescence imaging (Olympus LV200) of NanoLuc-tagged β_2_-adrenoceptors. MDA-MB-231^HM^ Nluc-β_2_AR cells treated with 400 nM furimazine substrate alone (upper panels) to detect luminescence in the absence of added fluorescent ligand using an open channel (20 s exposure time; 420 nm longpass filter; upper left panel) or a Cy5 channel (4 min exposure time; 600/50 nm bandpass filter; upper right panel) to detect BRET generated by binding of fluorescent ligand, when present. Middle and lower panels show images from cells treated with 50 nM Prop-BY630, in the presence (lower panels) or absence (middle panels) of unlabelled ICI 118551 (10 μM). Images shown were acquired with an open channel (middle and lower left panels) and the Cy5 channel (middle and lower right panels). Scale bar represents 50 μm. (C) BRET ratios obtained using bioluminescence imaging using ImageJ time series analyzer. Data show the mean and SE obtained in 3 independent experiments. **p < 0.01 compared with basal or in the presence of 10 μM ICI 118551 (one-way ANOVA with Tukey's multiple comparisons).

We began by generating an MDA-MB-231^HM^ cell line stably expressing the human β_2_-adrenoceptor with an N-terminal NanoLuc tag. Standard fluorescence confocal microscopy revealed some of the limitations of using a fluorescent probe without BRET (Figure 1A). Thus, following incubation with a fluorescent analog of the β-blocker propranolol (50 nM; Prop-BY630; Figure S1), fluorescence was detected by confocal imaging at both the cell membrane and in a discrete perinuclear region (Figure 1A; lower left panel). Cell membrane fluorescence was completely prevented by co-incubation with 10 μM unlabelled propranolol (Figure 1A; lower right panel), demonstrating specific binding of the ligand to the β_2_-adrenoceptors on the cell surface. Perinuclear labeling, however, was not displaced by co-incubation with 10 μM unlabelled propranolol indicating non-specific binding. Furthermore, only non-specific binding was detected in non-transfected MDA-MB-231^HM^ cells (Figure 1A; upper left panel). This demonstrates that although confocal microscopy can detect cell surface receptors using fluorescent ligands, interpretation of the fluorescence readout may be confounded in an *in vivo* setting by the extent of non-specific binding to non-receptor sites.

**Figure 2:**
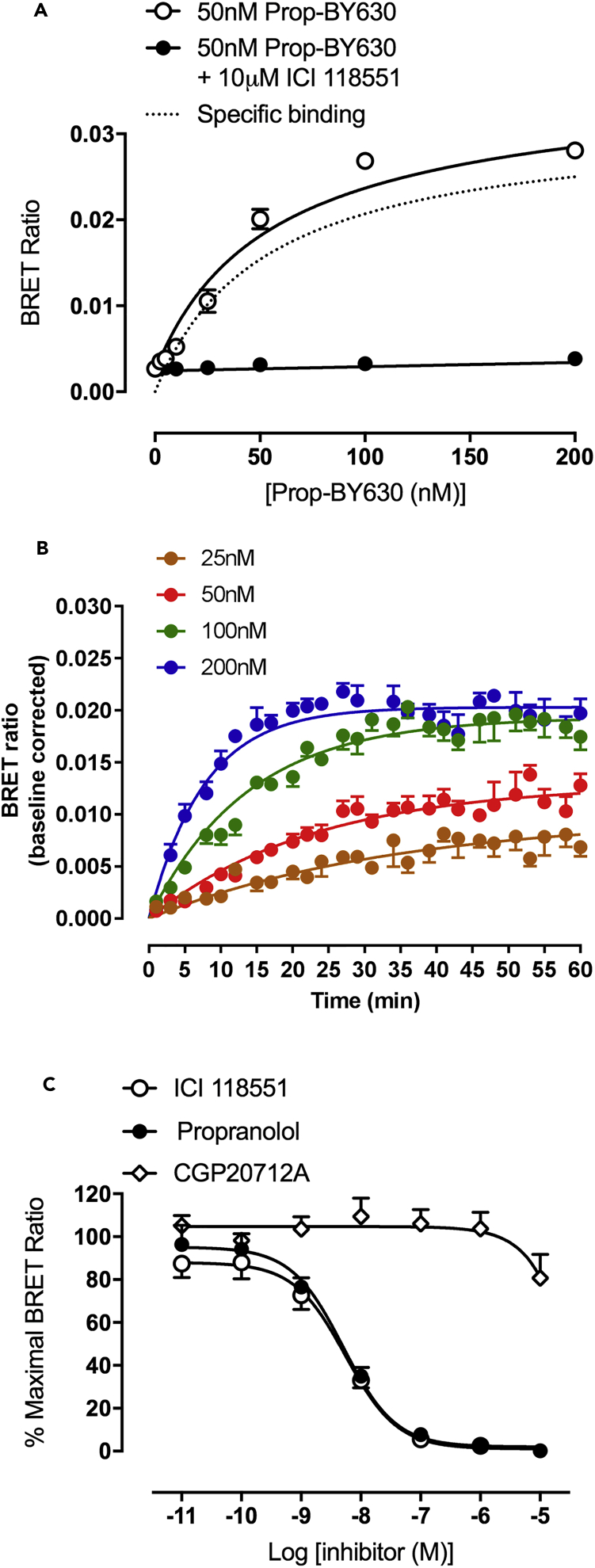
Quantitative Analysis of Ligand Binding to NanoLuc-Tagged Human β_2_-Adrenoceptors Expressed in MDA-MB-231^HM^ Cells Using NanoBRET (A) Binding of increasing concentrations of Propranolol-(β-Ala-β-Ala)-X-BODIPY630/650 (Prop-BY630) to NanoLuc-tagged human β_2_-adrenoceptors in MDA-MB-231^HM^ cells measured using a CLARIOstar plate reader. Non-specific binding was defined with 10 μM unlabelled ICI 118551. Data are mean ± SE from 6 separate experiments. Total (open circles) and non-specific binding (closed circles) curves were fitted simultaneously as described in the Transparent Methods. The dotted line shows the specific binding component derived from this analysis. (B) Real-time kinetic analyses of Prop-BY630 binding to NanoLuc-tagged human β_2_-adrenoceptors expressed in MDA-MB-231^HM^ cells using 25, 50, 100, and 200 nM fluorescent ligand. BRET ratios for kinetic studies have been baseline-corrected to specific binding (after subtraction of non-specific binding) at time 0. Data are mean ± SE of triplicate determinations in a representative experiment. Similar data were obtained in 4 additional experiments. (C) Inhibition of the specific binding of 50 nM Prop-BY630 to NanoLuc-tagged human β_2_-adrenoceptors in MDA-MB-231^HM^ cells by increasing the concentrations of ICI 118551, un-labelled propranolol, and CGP20712A. Data are mean ± SE from 5 separate experiments.

To determine if BRET with NanoLuc-tagged receptors in combination with fluorescent ligands had the specificity required to detect ligand-binding to GPCRs on the surface of cancer cells, we first used wide-field bioluminescence imaging to demonstrate the cell membrane location of the NanoLuc-tagged β_2_-adrenoceptor in MDA-MB-231^HM^ cells (Figure 1B; left panels). Addition of 50 nM Prop-BY630 allowed us to visualize the energy transfer from the N-terminal NanoLuc of the β_2_-adrenoceptor to the fluorescent ligand bound to the receptor via NanoBRET (detected in the Cy5 channel). This clearly revealed specific binding to cell surface β_2_-adrenoceptors that could be inhibited by a selective β_2_-antagonist ICI 118551 (10 μM; p < 0.001; Figures 1B and 1C). The BRET signal from this ligand-receptor interaction was also measured in a 96-well plate format using a CLARIOstar plate reader, and demonstrated that the specific binding detected by BRET increased with the concentration of the probe until all of the receptors on the cell surface had been occupied. This specific binding (the difference between total binding and non-specific binding) was clearly saturable (pK_D_ = 7.28 ± 0.07; n = 5; Figure 2A) and equally important for the future *in vivo* experiments; non-specific binding to non-receptor sites (obtained in the presence of 10 μM ICI 118551) was very low (Figure 2A; closed circles).

To determine the compatibility for *in vivo* studies, we also imaged the BRET signal using a whole-animal bioluminescence and fluorescence imaging system, IVIS Lumina II (Figure S2A), and obtained a comparable pK_D_ for fluorescent propranolol (7.26 ± 0.06, n = 5). This was consistent with the value obtained previously for this ligand in HEK293 cells (Stoddart et al., 2015). The BRET methodology also allowed us to determine the binding kinetics of fluorescent propranolol (k_on_ 5.4 ± 2.2 × 10^5^ M^−1^ min^−1^; k_off_ 0.025 ± 0.004 min^−1^; n = 5; Figure 2B). This confirmed that Prop-BY630 could bind rapidly to the β_2_-adrenoceptor, but once bound dissociated slowly (the reciprocal of k_off_ gives a residence time of 40 min), making it an ideal probe for *in vivo* use. Similar data for ligand binding kinetics/residence time were obtained in separate experiments using the IVIS system (Figure S2B).

To ensure that the BRET signal detected was confined to the β_2_-adrenoceptor, competition-binding experiments were undertaken with the β_2_-adrenoceptor-selective antagonist ICI 118 551, the β_1_-selective antagonist CGP 20712A (Gherbi et al., 2015), and the non-selective β-blocker propranolol (Figure 2C). These experiments yielded pK_i_ values that were consistent with literature values obtained previously for binding to β_2_-adrenoceptors (Stoddart et al., 2015; Figures 2C, S2C, and S2D; Table S1). Thus, the β_1_-selective antagonist CGP 20712A produced very little inhibition of fluorescent ligand binding at concentrations up to 10 μM (Figure 2C). These data confirm that this BRET proximity assay is exquisitely selective and only detects binding to NanoLuc-tagged β_2_-adrenoceptors expressed on the tumor cells. Furthermore, our data also confirmed that the optical properties of the IVIS system had the sensitivity to detect ligand binding to β_2_-adrenoceptors on cancer cells by BRET (Figure S2).

**Figure 3:**
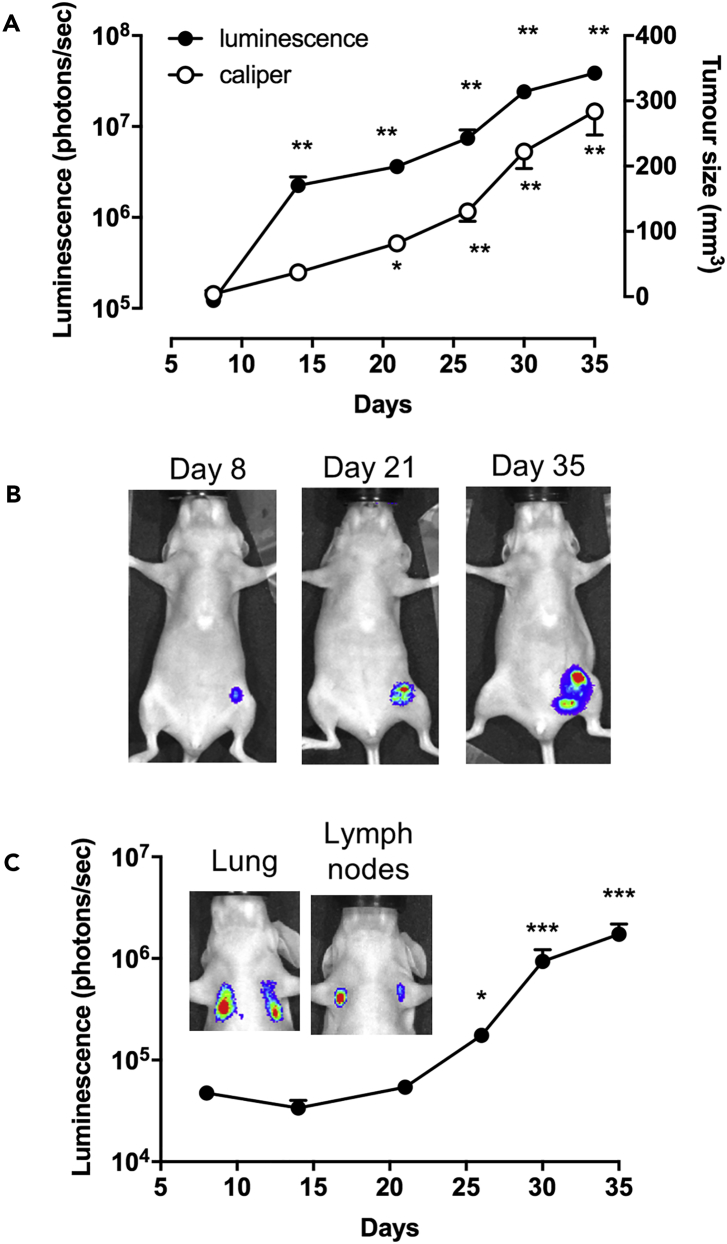
Whole-Animal Bioluminescence Imaging of NanoLuc-β_2_AR MDA-MB-231^HM^ Tumor Growth and Metastasis Development (A) Female BALB/c nu/nu mice (7-week-old) were injected in the fourth left mammary fat pad with 5 × 10^5^ MDA-MB-231^HM^ triple-negative human breast cancer cells that stably express the NanoLuc-tagged human β_2_-adrenoceptor. Tumor development was monitored by bioluminescence imaging (left y axis) 5 min after IV injection of the NanoLuc substrate furimazine (100 μL in PBS, *circa* 0.37 mg/kg) or by caliper measurements (right y axis) over 35 days. (B) Representative bioluminescence images of primary tumors. (C) Bioluminescence monitoring of the development of metastasis in lungs and axillary lymph nodes. Inset: representative images of lung and lymph node metastasis. Data were obtained from 11 mice and are expressed as mean ± SE. *p < 0.05, **p < 0.005, ***p < 0.0001 (two-way ANOVA with Tukey's multiple comparisons with respect to day 8 baseline). For luminescence measurements, the statistical analysis was applied to the log transformed values.

Previous work from our laboratory has shown that β_2_-adrenoceptors on MDA-MB-231^HM^ cells may play a significant role in the effect of stress on metastasis (Le et al., 2016, Sloan et al., 2010, Chang et al., 2016). Those studies used tumor cells that had been transfected with a cytosolic firefly luciferase marker to monitor primary tumor growth and metastasis in a mouse model of breast cancer (Le et al., 2016, Sloan et al., 2010, Chang et al., 2016). Cytosolic NanoLuc luciferase has also been used to monitor cancer progression in living animals (Stacer et al., 2013). In contrast, here we used receptor-specific NanoLuc bioluminescence to localize *in vivo* tumor cells that specifically express β_2_-adrenoceptors. In mice injected with tumor cells into the mammary fat pad, luminescence intensity from NanoLuc-β_2_-adrenoceptors (photons/sec) increased from 8 days after tumor cell injection (Figures 3A and 3B) and correlated with primary tumor size determined by caliper (mm^3^; Pearson correlation: p < 0.0001, R^2^ = 0.560; Figure 3A). Metastatic tumors containing cells expressing β_2_-adrenoceptors appeared in the lung and axillary lymph nodes later in tumor development (Figure 3C).

To detect ligand binding *in vivo* by BRET we injected Prop-BY630 (0.1 mg/kg) directly into the tumor (intratumoral [IT]) and used the IVIS Lumina II imaging system to monitor the red fluorescence emission from the fluorescent ligand relative to the blue luminescence donor emission from the NanoLuc-labeled β2-adrenoceptors. This ratiometric approach determines the level of specific ligand binding independently of the number of cells expressing NanoLuc-β_2_-adrenoceptors present in the tumor. Thus, regardless of the slight variation in tumor burden between mice, we were able to compare how well Prop-BY630 interacted with the target β_2_-adrenoceptor on tumor cells. Preliminary experiments established that addition of 0.1 mg/kg Prop-BY630 delivered directly into the tumor reached a steady plateau BRET ratio, significantly above baseline values (n = 7; p < 0.001; two-way ANOVA), within *circa* 15 min of administration of the fluorescent ligand (Figure S3).

**Figure 4:**
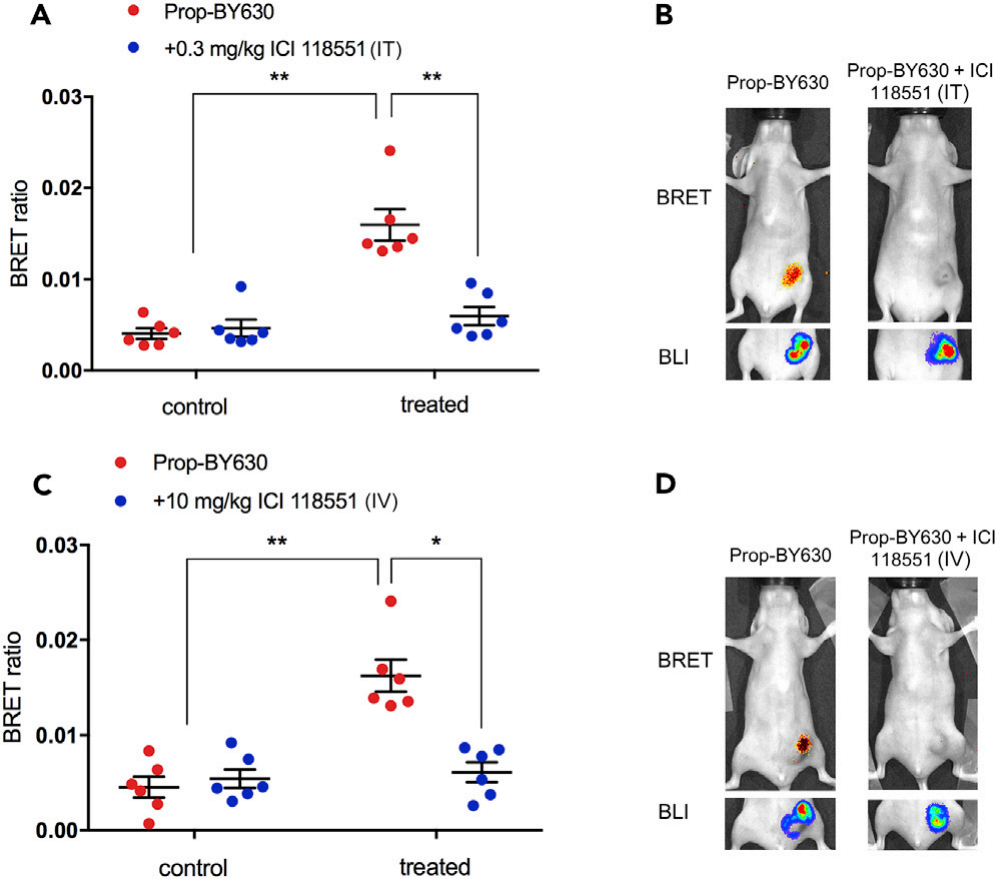
NanoBRET to Monitor Specific Ligand-Receptor Binding *In Vivo* in Primary MDA-MB-231^HM^ Tumors (A) Female BALB/c nu/nu mice (7-week-old) were injected in the fourth left mammary fat pad with 5 × 10^5^ MDA-MB-231 triple-negative human breast cancer cells stably expressing Nluc-β_2_AR. Once tumor size reached >200 mm^3^; BRET ratios were determined from mice administered fluorescent propranolol-(β-Ala-β-Ala)-X-BODIPY630/650 alone (Prop-BY630 at 0.1 mg/kg intra-tumor, IT; red circles) or from mice receiving both Prop-BY630 (0.1 mg/kg IT) and 0.3 mg/kg ICI 118551 (IT; blue circles). ICI 118551 was administered 45 min before the fluorescent propranolol. Control represents measurements taken 5 min after IV injection (in 100 μL; *circa* 0.37 mg/kg) of the furimazine substrate 24 hr before administration of any β_2_-adrenoceptor-directed ligands. BRET measurements of ligand binding were made 1 hr after injection of Prop-BY630 (treated condition). Furimazine substrate was injected IV 5 min before mice were imaged. Data represent mean ± SE of 6 mice in each group. **p < 0.0001 (two-way ANOVA with Tukey's multiple comparison test) compared with both control datasets and the 1 hr treatment with Prop-BY630. (B) Representative images showing single mice exposed to Prop-BY630 only (left panel) or to 0.3 mg/kg (IT) ICI 118551 and 0.1 mg/kg (IT) Prop-BY630 (right panel). Upper panels shown in (B) show BRET images and lower panels show the total bioluminescence (BLI) from the NanoLuc. (C) BRET ratios determined from mice administered with fluorescent ligand alone (Prop-BY630 0.1 mg/kg IT; red circles) or from mice receiving both Prop-BY630 (0.1 mg/kg IT) and 10 mg/kg ICI 118551 (IV; blue circles). ^∗∗^p<0.0001 (two-way ANOVA with Tukey's multiple comparison test) compared with both control datasets. ^∗^p<0.001 compared to 1h treatment with Prop-BY630. (D) Representative images showing mice exposed to 0.1 mg/kg Prop-BY630 (IT) alone or to 10 mg/kg ICI 118551 (IV) and 0.1 mg/kg Prop-BY630. Data represent mean ± SE of 6 mice in each group.

In a separate experiment with a crossover design, administration of the fluorescent ligand significantly increased the BRET ratios for each mouse (measured after 1 hr), demonstrating detection of specific ligand binding to β_2_-adrenoceptors (Figures 4A and 4C; p < 0.0001). Receptor engagement by the fluorescent ligand was prevented by pre-treating mice with the β_2_-selective antagonist ICI 118551, delivered either directly into the tumor (0.3 mg/kg IT p < 0.0001; Figures 4A and 4B) or through the tail vein (10 mg/kg intravenously [IV] p < 0.001; Figures 4C and 4D), demonstrating that the assay can be used to quantify the extent to which unlabeled drugs (e.g., ICI 118551) can engage with their molecular target when delivered directly into the tumor or through the circulation.

**Figure 5:**
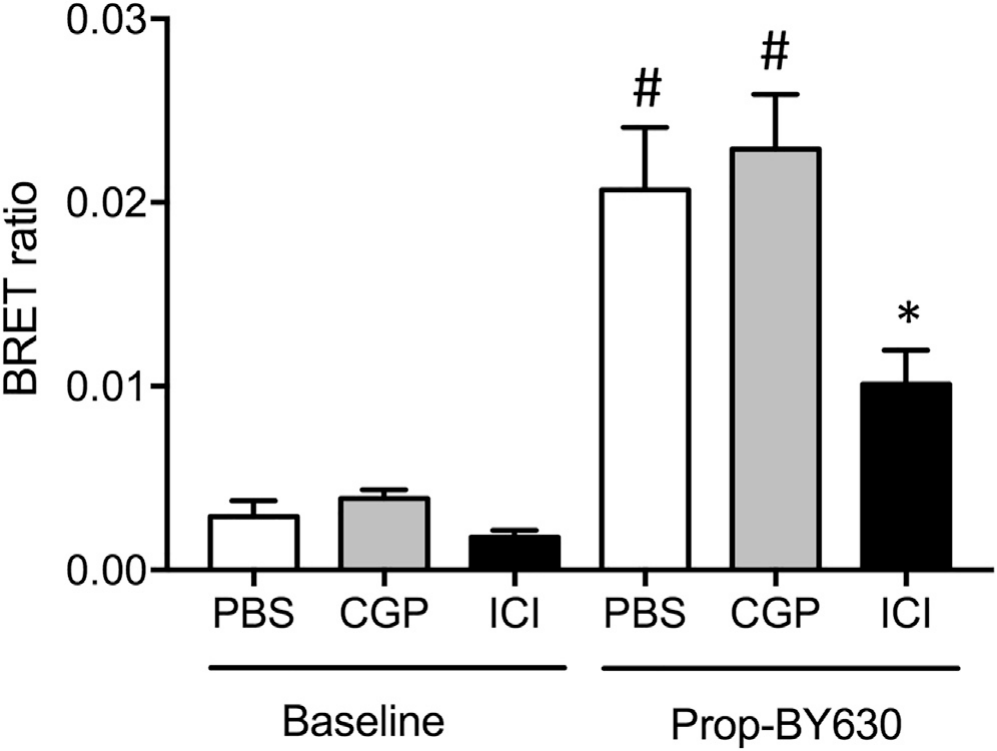
Effect of Selective β_1_- and β_2_-Adrenoceptor Antagonists on Prop-BY630 Binding to MDA-MB-231^HM^ Cells *In Vivo* Drug-receptor engagement in the primary tumor region was investigated using 0.1 mg/kg (IT) Prop-BY630 in mice treated with the β_2_-selective antagonist ICI 118551 or the β_1_-selective antagonist CGP20712A. At 45 min before fluorescent ligand injection, mice were administered (IV) with either PBS (100 μL), CGP201712A (100 μL; 10 mg/kg in PBS), or ICI 118551 (100 μL; 1 mg/kg in PBS). Data represent mean ± SE of 6 mice in each group. Measurements were made 1 hr after administration of Prop-BY630. Baseline measurements were obtained on the previous day in each mouse when furimazine was administered via the tail vein, but Prop-BY630 was not injected into the tumor. *p < 0.05 or ^#^p < 0.001 with respect to the baseline (t = 0) signal in each group. *p < 0.05 versus control Prop-BY630 binding (1 hr PBS). One-way ANOVA with Tukey's posthoc tests.

Drug-receptor engagement in the primary tumor region was also investigated for a lower dose of ICI 118551 administered IV (1 mg/kg), as well as for the β_1_-selective antagonist CGP20712A (10 mg/kg IV; 100 μL in PBS) (Figure 5). These experiments demonstrated that pre-treatment with CGP20712A produced no significant attenuation of the specific binding of Prop-BY630 to β_2_-adrenoceptors on MDA-MB-231^HM^ tumors *in vivo,* whereas a 10-fold lower dose of the β_2_-selective antagonist ICI 118551 produced a significant inhibition (p < 0.05; Figures 5; compare Figure 4B). These data demonstrate the ability of this assay to accurately report target engagement *in vivo*. The data obtained with ICI 188551 indicate that 1 mg/kg (IV) is a dose that achieves roughly 50% target engagement of tumor cell β_2_-adrenoceptors within this solid breast cancer tumor model (Figure 5).

**Figure 6:**
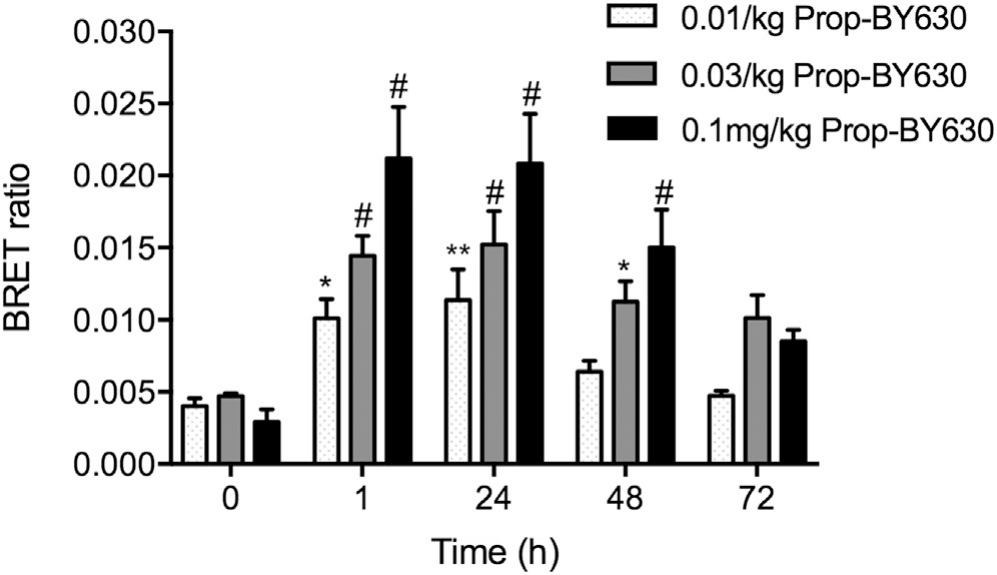
Dose-Dependent Binding of Prop-BY630 to β_2_-Adrenoceptors and Subsequent Ligand Dissociation *In Vivo* To monitor fluorescent ligand dissociation over time in the primary tumors, mice were administered with 3 different doses of propranolol-(β-Ala-β-Ala)-X-BY630/650 (0.01, 0.03, or 0.1 mg/kg; IT). At 1, 24, 48, and 72 hr after fluorescent ligand injection, mice were injected with furimazine substrate (IV, 100 μL in PBS, *circa* 0.37 mg/kg) and imaged 5 min later using the IVIS Lumina II camera system. Imaging was performed by capturing sequential luminescence (open channel, 30 s exposure time) and fluorescence (Cy5.5 channel, 5 min exposure times) images. All mice were also imaged on the day before fluorescent ligand injection following an IV injection of furimazine to determine luminescence (and BRET) baseline (t = 0). Data represent mean ± SE of 6 mice in each group. *p < 0.05. **p < 0.01 or ^#^p < 0.001 compared with corresponding time 0 controls. Two-way ANOVA with repeated measures and Dunnett multiple comparisons.

Finally, to establish the sensitivity of the assay to different doses of the fluorescent ligand, we monitored *in viv*o ligand-binding BRET responses following addition of 0.01, 0.03, or 0.1 mg/kg of Prop-BY630 and quantified the *in vivo* receptor residence times over 72 hr (Figure 6). These experiments demonstrated significant ligand binding that could be detected by BRET with all 3 doses of the fluorescent ligand in a dose-dependent manner (Figure 6). Furthermore, binding of each of the 3 doses of Prop-BY630 to tumor β_2_-adrenoceptors was maintained for at least 48 hr after initial drug application (Figure 6). These data are consistent with the slow off-rate kinetics of Prop-BY630 from the β2-adrenoceptor observed *in vitro* (Figure 2) and also suggest that the drug is not rapidly cleared from the tumor environment.

The close proximity requirements (10 nm) for BRET to occur between the donor NanoLuc on the N-terminus of human β_2_-adrenoceptors on tumor cells and the receptor-bound Prop-BY630 therefore provides a very sensitive and selective ligand binding assay to monitor receptor target engagement in tumors *in vivo*. As a consequence, the BRET readout should not be influenced by fluorescent ligand binding to neighboring endogenous receptors on other cell types (e.g., vascular and immune cells) within the tumor microenvironment. We therefore believe that this approach has significant advantages in specificity over other *in vivo* imaging modalities such as positron emission tomography (Hazan et al., 2017) for the study of specific receptor target engagement in tumors.

In summary, the present study has shown that ligand binding to a GPCR can be monitored *in vivo* using BRET. Here we have used triple-negative human breast cancer cells expressing human β_2_-adrenoceptors tagged with the bioluminescence protein NanoLuc to demonstrate that parenterally applied drugs can access receptors on tumor cells in a mouse model of breast cancer. This *in vivo* NanoBRET method will be widely applicable to monitor target engagement in animal models for other cell surface receptors such as receptor tyrosine kinases (Kilpatrick et al., 2017) and for intracellular kinases (Robers et al., 2015).

### Limitations of Study

The resolution of *in vivo* nanoBRET was limited in the present study to the detection of established tumors including macro-metastases. Future development of fluorescent ligands with increased water solubility should increase the stability in plasma and improve the detection of IV-administered probes. The development of new nanoluciferase substrates should also improve the *in vivo* detection of resonance energy transfer.

## Methods

All methods can be found in the accompanying Transparent Methods supplemental file.

## Supplemental Information

Document S1. Transparent Methods, Figures S1–S3, and Table S1
