# Supplementary material for: Visualizing Ligand Binding to a GPCR In Vivo Using NanoBRET
Source: iScience. 2018 Aug 11;6:280–8. doi: 10.1016/j.isci.2018.08.006 (PMC6137713; doi:10.1016/j.isci.2018.08.006)
Supplement: Document S1. Transparent Methods, Figures S1–S3, and Table S1 [file mmc1.pdf]

ISCI, Volume *6*

Supplemental Information

Visualizing Ligand Binding to a GPCR *In Vivo* Using NanoBRET

Diana C. Alcobia, Alexandra I. Ziegler, Alexander Kondrashov, Eleonora Comeo, Sarah Mistry, Barrie Kellam, Aeson Chang, Jeanette Woolard, Stephen J. Hill, and Erica K. Sloan

Supplemental Information

Visualising ligand-binding to a GPCR *in vivo* using nanoBRET.

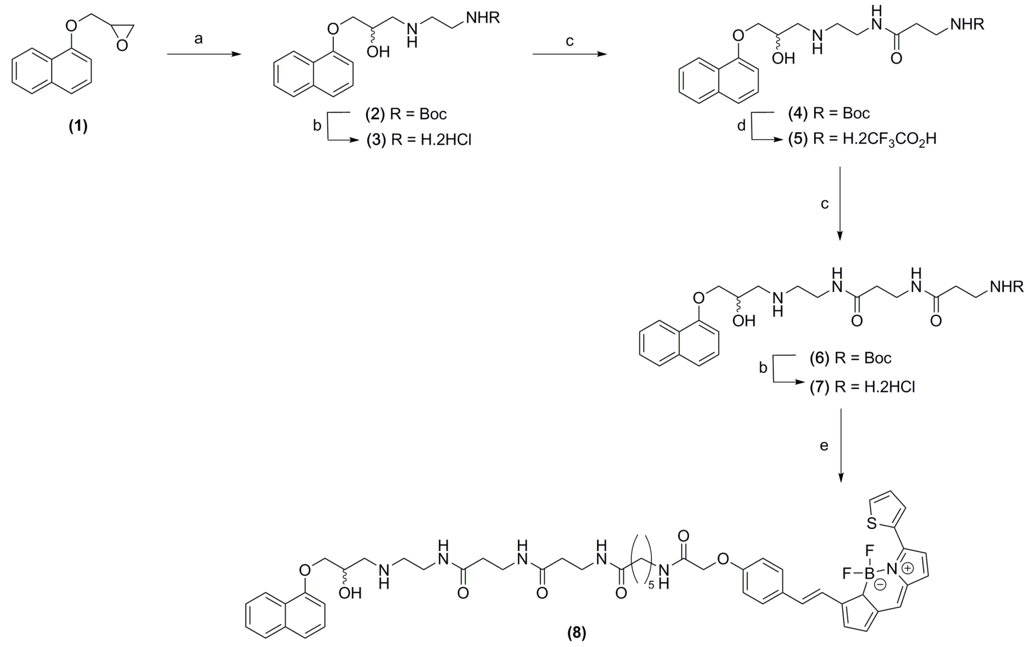

Figure S1. Reaction scheme for the synthesis of Propranolol-(β-Ala-β-Ala)-X-BODIPY630/650 (Compound 8 in the figure; Prop-BY630). Related to Figure 1. Reagents and conditions: (a) tert-Butyl-2-aminoethyl carbamate (Boc), DMF/water (9:1), 85°C, 16h, 39%; (b) 4M HCl in dioxane, 5h, 29-100%; (c) Boc-β-Ala-OH, HBTU, DIPEA, DMF, room temperature, 35-41%; (d) TFA, DCM, room temperature, 2h, 100%; (e) BODIPY630/650-X-SE, DIPEA, DMF, room termperature, 3h, 91%. Abbreviations: BODIPY 630/650-X-SE, 6-(((4,4-difluoro-5-(2-thienyl)-4-bora-3a,4a-diaza-s-indacene-3-yl)-styryloxy)acetyl)aminohexanoic acid succinimidyl ester; DCM, dichloromethane; DIPEA, diisopropylethylamine; DMF, *N,N*-dimethylformamide; HBTU, *N,N,N',N'*-Tetramethyl-*O*-(1*H*-benzotriazol-1-yl)uronium hexafluorophosphate; TFA, trifluoroacetic acid.

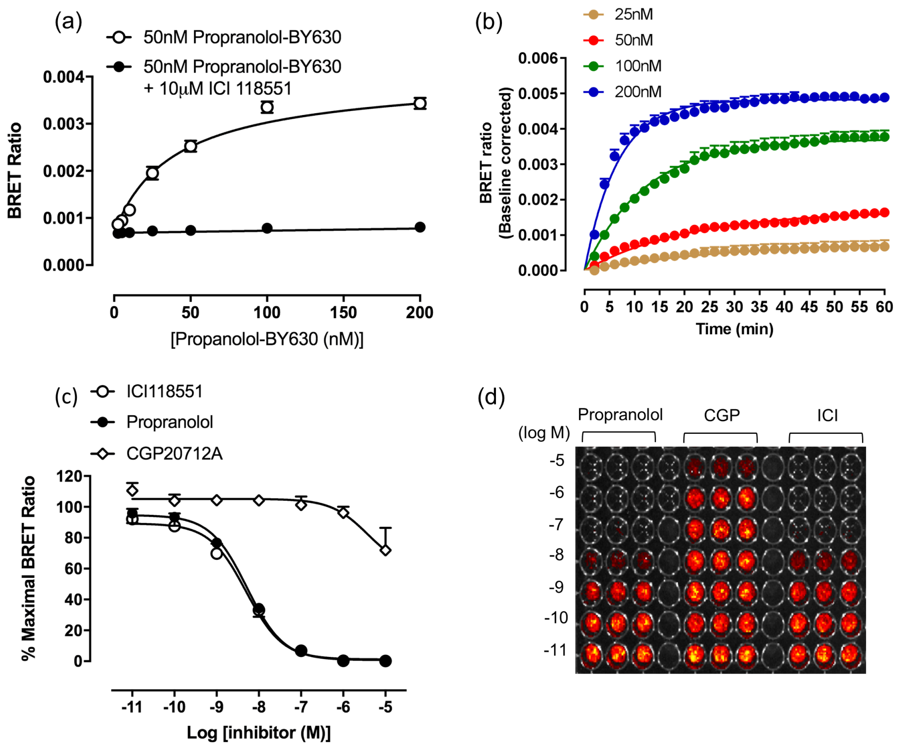

Figure S2. Quantitative analysis of ligand-binding to NanoLuc-tagged human β_2-adrenoceptors expressed in MDA-MB-231^{HM} cells using BRET imaging with the IVIS lumina II system. Related to Figure 2. (a) Binding of increasing concentrations of Propranolol-(β-Ala-β-Ala)-X-BODIPY630/650 (Prop-BY630) to NanoLuc-tagged human β_2-adrenoceptors in MDA-MB-231^{HM} cells measured using the IVIS lumina II camera system. Non-specific binding was defined with 10μM unlabelled ICI 118551. Data are mean ± S.E. of triplicate determinations in a representative experiment. Similar data were obtained in four further experiments. (b) Real time kinetic studies of Prop-BY630 binding to NanoLuc-tagged human β_2-adrenoceptors expressed in MDA-MB-231^{HM} cells using 25, 50, 100 and 200nM fluorescent ligand. Data are mean ± S.E. of triplicate determinations in a representative experiment. BRET ratios for kinetic studies have been baseline-corrected to the specific binding (after subtraction of non-specific binding) BRET ratio obtained at time zero. Similar data were obtained in two further experiments. (c) Inhibition of the specific binding of 50nM Prop-BY630 to NanoLuc-tagged human β_2-adrenoceptors in MDA-MB-231^{HM} cells by increasing concentrations of ICI 118551, propranolol and CGP20712A. Data are mean ± S.E. from four separate experiments. (d) Representative plate image obtained using the IVIS system (using the Cy5.5 emission channel) for the competition binding assay shown in (c) with ICI 118551 (ICI), propranolol and CGP 20712A (CGP).

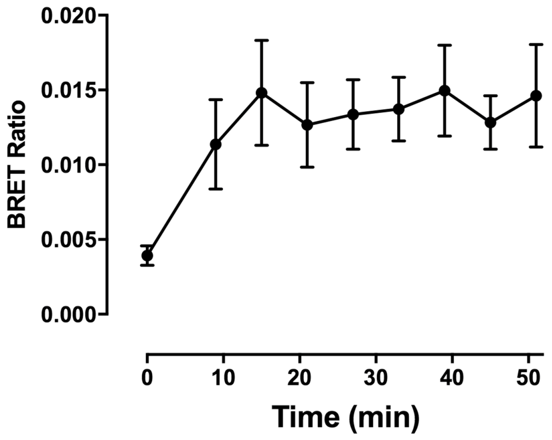

Figure S3. Time course of the binding of Prop-BY630 to β_2-adrenoceptors in vivo. Related to Figure 4. Mice were injected with 0.1 mg/kg Propranolol-(β-Ala-β-Ala)-X-630/650 (in 50μl of PBS) directly into the primary tumour (intratumoral; i.t). Immediately following this, mice were injected with 100μL 1:20 dilution furimazine substrate via the tail vein (i.v., 100μL diluted in PBS; *circa* 0.37 mg/kg) and imaged using the IVIS lumina II camera system. Mice were kept under 2-3% isoflurane anaesthesia during injections and imaging. Sequential luminescence (open channel, 30 sec exposure time) and fluorescence (Cy5.5. channel using 660/20nm bandpass filter; 5 min exposure time) images were taken every 6 min for a total time of 49 min. Images were acquired from 1 min after fluorescent ligand injection. Values show mean $\pm$ S.E from seven mice. 2-Way ANOVA confirmed that there was a significant time-dependent effect ($p<0.001$).

Table S1. Table of pK_i values obtained from competition experiments using the CLARIOstar plate reader or the IVIS imaging camera system. Related to Figure 2.

Unlabelled Ligands	CLARIOStar<br><br>pK_i (mean ± S.E)	n	IVIS<br><br>pK_i (mean ± S.E)	n
ICI 118551	8.55 ± 0.05	5	8.55 ± 0.02	4
Propranolol	8.68 ± 0.17	5	8.69 ± 0.18	4

Equilibrium binding parameters obtained from inhibition of the specific binding of 50nM Prop-BY630 to NanoLuc-tagged human β_2-adrenoceptors in MDA-MB-231^{HM} cells by increasing concentrations of ICI 118551 and un-labelled propranolol. Data are mean ± S.E. from n separate experiments.

TRANSPARENT METHODS

Experimental Procedures.

Synthesis of Propranolol-β-ala-β-ala-BODIPY 630/650.

Propranolol-(β-alanine,β-alanine)-X-BODIPY630/650 (Stoddart et al., 2015; Soave et al., 2016) was synthesised as described in the reaction scheme in Supplementary Figure S1.

Chemicals and solvents of analytical and HPLC grade were purchased from commercial suppliers and used without further purification. BODIPY-630/650-X-SE was purchased from Molecular Probes (Thermo Fisher Scientific). All reactions were carried out at ambient temperature unless otherwise stated. Reactions were monitored by thin-layer chromatography on commercially available silica pre-coated aluminium-backed plates (Merck Kieselgel 60 F254). Visualisation was under UV light (254 nm and 366 nm), followed by staining with ninhydrin or $KMnO_4$ dips. Flash column chromatography was performed using silica gel 60, 230-400 mesh particle size (Sigma Aldrich). NMR spectra were recorded on a Bruker-AV 400. ^{1}H spectra were recorded at 400.13 Hz and ^{13}C NMR spectra at 101.62 Hz. All ^{13}C NMR are ^{1}H broadband decoupled. Solvents used for NMR analysis (reference peaks listed) were $CDCl_3$ supplied by Cambridge Isotope Laboratories Inc., (δ_H = 7.26 ppm, δ_C = 77.16) and CD_3OD supplied by VWR (δ_H = 3.31 ppm and δ_C = 49.00). Chemical shifts (δ) are recorded in parts per million (ppm) and coupling constants are recorded in Hz. The following abbreviations are used to described signal shapes and multiplicities; singlet (s), doublet (d), triplet (t), quadruplet (q), broad (br), dd (doublet of doublets), ddd (double doublet of doublets), dtd (double triplet of doublets) and multiplet (m). Spectra were assigned using appropriate COSY and HSQC experiments. Processing of the NMR data was carried out using the NMR software Topspin 3.0. LC-MS spectra were recorded on a Shimadzu UFLCXR system coupled to an Applied Biosystems API2000 and visualised at 254 nm (channel 1) and 220 nm (channel 2). LC-MS was carried out using a Phenomenex Gemini-NX C18 110A, column (50 mm × 2 mm x 3 µm) at a flow rate 0.5 mL/min over a 5 min period (Method A). All high resolution mass spectra (HRMS) were recorded on a Bruker microTOF mass spectrometer using MS electrospray ionization operating in positive ion mode. RP-HPLC was performed on a Waters 515 LC system and monitored using a Waters 996 photodiode array detector at wavelengths between 190 and 800 nm. Spectra were analysed using Millenium 32 software. Semi-preparative HPLC was performed using YMC-Pack C8 column (150 mm × 10 mm × 5 µm) at a flow rate of 5.0 mL/min using a gradient method of 40-95% B over 15 minutes (Solvent A = 0.01% formic acid in H_2O, solvent B = 0.01% formic acid in CH_3CN (Method B)). Analytical RP-HPLC was performed using a YMC-Pack C8 column (150 mm × 4.6 mm × 5 µm) and a Phenomenex Gemini NX-C18 column

(250 mm × 4.6 mm × 5 μm) at a flow rate of 1.0 mL/min. Final products were one single peak and >95% pure. The retention time of the final product is reported using a gradient method of 5-95% solvent B in solvent A over 25 minutes. (Solvent A = 0.01% formic acid in H_2O, solvent B = 0.01% formic acid in CH_3CN (Method C)).

Abbreviations; BODIPY 630/650-X-SE, 6-(((4,4-difluoro-5-(2-thienyl)-4-bora-3a,4a-diaza-s-indacene-3-yl)-styryloxy)acetyl)aminohexanoic acid succinimidyl ester; DCM, dichloromethane; DIPEA, diisopropylethylamine; DMF, *N,N*-dimethylformamide; HBTU, *N,N,N′,N′*-Tetramethyl-*O*-(1*H*-benzotriazol-1-yl)uronium hexafluorophosphate; TFA, trifluoroacetic acid.

(±)-2-((Naphthalen-1-yloxy)methyl)oxirane (1)

The title compound was synthesised as previously described in the literature (Baker et al., 2011).

(±)-*tert*-Butyl 2-(2-hydroxy-3-(naphthalen-1-yloxy)propylamino)ethylcarbamate (2)

A solution of (±)-2-((naphthalen-1-yloxy)methyl)oxirane **(1)** (0.241 g, 1.20 mmol) and *tert*-butyl-2-aminoethylcarbamate (0.482 g, 3.01 mmol) in a mixture of DMF/water 9:1 (6 mL), was heated at 85°C for 16 hours. The solvent was removed under reduced pressure and the crude product was purified by column chromatography on silica (3% 1M NH_3 in MeOH/DCM). The title compound was afforded as an off-white solid (0.168g, 39%). ^{1}H NMR (400 MHz, $CDCl_3$) δ 8.30 – 8.18 (m, 1H), 7.84 – 7.76 (m, 1H), 7.54 – 7.41 (m, 3H), 7.36 (dd, *J* = 8.3, 7.5 Hz, 1H), 6.82 (dd, *J* = 7.6 Hz, *J* = 1.0 Hz, 1H), 4.95 (t, *J* = 5.3 Hz, 1H), 4.28 – 4.22 (m, 1H), 4.22-4.11 (m, 2H) 3.34 – 3.22(m, 2H) 3.00 (dd, *J* = 12.3, 3.7 Hz, 1H), 2.92 (dd, *J* = 12.2, 7.5 Hz, 1H), 2.83 (ddd, *J* = 6.2, 1.5 Hz, 2H), 1.44 (s, 9H). ^{13}C NMR (101 MHz, $CDCl_3$) δ 156.41, 154.36, 134.63, 127.68, 126.60, 125.94, 125.65, 125.45, 121.91, 120.85, 105.08, 70.62, 68.59, 53.57, 51.90, 49.52, 28.55. LC-MS *m/z* calc. for $C_{20}H_{29}N_2O_4$ $[MH]^+$; 361.2, found; 361.2, t_R = 2.29 min.

(±)-2-(2-Hydroxy-3-(naphthalen-1-yloxy)propyl-amino)ethylamine dihydrochloride (3)

To a solution of (±)-*tert*-butyl 2-(2-hydroxy-3-(naphthalen-1-yloxy)propyl-amino)ethylcarbamate (**2**) (0.063 g, 0.18 mmol) in Et_2O (0.5 mL) 4M HCl in dioxane (0.5 mL) was added and the mixture was stirred for 5 hours. The solvent was removed under reduced pressure to afford the title compound as a pale pink solid (0.060 g, 100%). ^{1}H NMR (400 MHz, CD_3OD) δ 8.36 – 8.26 (m, 1H), 7.86 – 7.78 (m, 1H), 7.58 – 7.43 (m, 3H), 7.39 (dd, *J* = 8.3, 7.6 Hz, 1H), 6.95 (dd, *J* = 7.6 Hz, 1H), 4.64-4.41 (m, *J* = 9.8 Hz, 1H), 4.27 (dd, *J* = 10.0, 5.0 Hz, 1H), 4.21 (dd, *J* = 10.0, 5.5 Hz, 1H), 3.57 – 3.45 (m, 3H), 3.43 – 3.35 (m, 3H). ^{13}C NMR (101 MHz, CD_3OD) δ 155.35, 136.08, 128.59, 127.51, 126.92, 126.79, 126.28, 122.80,

121.98, 106.16, 71.05, 66.84, 51.97, 45.82, 36.69. LC-MS *m/z* calc. for $C_{15}H_{21}N_2O_2$ $[MH]^+$; 261.2, found; 261.2, t_R = 0.62 min.

(±)-*tert*-Butyl(3-((2-((2-hydroxy-3-(naphthalen-1-yloxy)propyl)amino)ethyl)amino)-3-oxopropyl)carbamate (4)

To Boc-β-Ala-OH (0.090 g, 0.47 mmol) in DMF (2 mL) HBTU (0.213 g, 0.56 mmol) and DIPEA (0.302 g, 2.34 mmol) were added. The mixture was stirred for 10 minutes and (±)-2-(2-Hydroxy-3-(naphthalen-1-yloxy)propyl-amino)ethylamine dihydrochloride (**3)** (0.060 g, 0.18 mmol) in DMF (1.5 mL) was added. After stirring for 2 hours the reaction mixture was partitioned between EtOAc and a 1:1 mixture of sat. $NaHCO_{3\,(aq)}$/water. The aqueous layer was washed with EtOAc x2 and the combined organic phases were dried over $MgSO_4$, filtered and concentrated under reduced pressure. The crude material was purified by column chromatography (10% 1M NH_3 in MeOH/DCM) to afford the title compound as a colourless oil (0.072 g, 35%). ^{1}H NMR (400 MHz, $CDCl_3$) δ 8.27 – 8.18 (m, 1H), 7.85 – 7.73 (m, 1H), 7.58 – 7.41 (m, 3H), 7.39-7.31 (m, Hz, 1H), 6.80 (d, *J* = 7.5 Hz, 1H), 6.56 (s, 1H), 5.30 (s, 1H), 4.45-4.25 (m, 1H), 4.25 – 4.08 (m, 2H), 3.48-3.41 (m, 2H), 3.41-3.33 (m, 2H), 3.12 -2.78 (m, 4H), 2.43-2.34 (m, 2H), 1.41 (s, 9H). ^{13}C NMR (101 MHz, $CDCl_3$) δ 172.52, 154.21, 134.63, 127.71, 126.65, 125.95, 125.58, 125.51, 121.85, 120.97, 105.11, 70.43, 51.78, 50.99, 48.95, 36.71, 28.54. LC-MS *m/z* calc. for $C_{23}H_{34}N_3O_5$ $[MH]^+$; 432.2, found; 432.2, t_R = 2.25 min.

(±)-3-((2-((2-Hydroxy-3-(naphthalen-1-yloxy)propyl)amino)ethyl)amino)-3-oxopropylamine ditrifluoroacetate (5)

To a solution of (±)-*tert*-butyl(3-((2-((2-hydroxy-3-(naphthalen-1-yloxy)propyl)amino)ethyl)amino)-3-oxopropyl)carbamate **(4)** (0.072 g, 0.17 mmol) in DCM (0.5 mL), TFA (0.5 mL) was added. The mixture was stirred at room temperature for 2 hours and the solvent was then removed under reduced pressure to afford the title compound as an off-white viscous solid which was used directly in the next step (0.054 g, 100%). LC-MS *m/z* calc. for $C_{23}H_{34}N_3O_5$ $[MH]^+$; 332.2, found; 322.1. LCMS t_R = 0.98 min.

(±)-*tert*-Butyl-(3-((3-((2-((2-hydroxy-3-(naphthalen-1-yloxy)propyl)amino)ethyl)amino)-3-oxopropyl)amino)-3-oxopropyl)carbamate (6)

The synthesis of the title compound **6** was carried out as described for (±)-*tert*-butyl(3-((2-((2-hydroxy-3-(naphthalen-1-yloxy)propyl)amino)ethyl)amino)-3-oxopropyl)carbamate **(4)**, using (±)-3-((2-((2-hydroxy-3-(naphthalen-1-yloxy)propyl)amino)ethyl)amino)-3-oxopropylamine ditrifluoroacetate **(5)**. The title compound was afforded as an off-white viscous solid (0.034 g, 41%). ^{1}H NMR (400 MHz, CD_3OD) δ 8.34 – 8.25 (m, 1H), 7.85 – 7.76 (m, 1H), 7.56 – 7.44 (m, 3H), 7.43 – 7.35 (m, 1H), 6.94 (dd, *J* = 7.6, 1.0 Hz, 1H), 4.53 - 4.43 (m, 1H), 4.26 (dd, *J* = 9.9, 5.0 Hz, 1H), 4.20 (dd, *J* = 9.9, 5.6 Hz, 1H), 3.58 – 3.41 (m, 4H),

3.36 – 3.34 (m, 2H), 3.29 – 3.24 (m, 4H), 2.44-2.26 (m, 4H). ^{13}C NMR (101 MHz, CD_3OD) δ 180.17, 155.35, 155.01, 136.08, 135.11, 130.26, 128.58, 127.51, 127.51, 126.92, 126.79, 126.28, 125.33, 122.79, 121.98, 118.74, 118.31, 106.16, 71.05, 66.84, 51.97, 45.82, 36.69. LC-MS *m/z* calc. for $C_{26}H_{39}N_4O_6$ $[MH]^+$; 503.3, found; 503.3, t_R = 2.25 min.

(±)-3-((3-((2-((2-Hydroxy-3-(naphthalen-1-yloxy)propyl)amino)ethyl)amino)-3-oxopropyl)amino)-3-oxopropylamine dihydrochloride (7)

Deprotection of (±)-*tert*-butyl-(3-((3-((2-((2-hydroxy-3-(naphthalen-1-yloxy)propyl)amino)ethyl)amino)-3-oxopropyl)amino)-3-oxopropyl)carbamate **(6)** (0.082 g, 0.16 mmol) was carried out as described for the synthesis of (±)-2-(2-Hydroxy-3-(naphthalen-1-yloxy)propyl-amino)ethylamine dihydrochloride **(3)**. The title compound was afforded as a white solid (0.019 g, 29%). ^{1}H NMR (400 MHz, CD_3OD) δ 8.35-8.26 (m, 1H), 7.92 – 7.74 (m, 1H), 7.57 – 7.43 (m, 3H), 7.39 (dd, *J* = 8.3, 7.5 Hz, 1H), 6.95 (d, *J* = 7.6 Hz, 1H), 4.48 (dtd, *J* = 9.8, 4.9, 3.0 Hz, 1H), 4.33 – 4.13 (m, 2H), 3.77 – 3.70 (m, 1H), 3.69 – 3.63 (m, 2H) 3.61 – 3.55 (m, 2H), 3.48 (t, *J* = 6.4 Hz, 3H), 3.41-3.32 (m, 1H), 3.18 (t, *J* = 6.5 Hz, 2H), 2.60 (t, *J* = 6.5 Hz, 2H), 2.45 (t, *J* = 6.5 Hz, 2H), 1.41 – 1.35 (m, 1H). ^{13}C NMR (101 MHz, CD_3OD) δ 175.66, 172.33, 155.40, 136.05, 128.54, 127.49, 126.93, 126.81, 126.28, 122.88, 121.90, 106.16, 71.15, 68.13, 66.86, 51.53, 37.16, 37.11, 37.06, 36.76, 33.00, 32.32. LC-MS *m/z* calc. for $C_{21}H_{31}N_4O_4$ $[M+H]^+$; 403.3, found; 403.3, t_R = 0.91 min.

***N*-(3-((3-((2-((2-Hydroxy-3-(naphthalen-1-yloxy)propyl)amino)ethyl)amino)-3-oxopropyl)amino)-3-oxopropyl)-6-((4-(2-(4,4-difluoro-4,4a-dihydro-5-(thiophen-2-yl)-4-bora-3a,4a-diaza-s-indacene-3-yl)vinyl)phenoxy)acetamido)-hexanamide (8, propranolol-β-Ala-β-Ala-X-BODIPY630/650)**

To (±)-3-((3-((2-((2-hydroxy-3-(naphthalen-1-yloxy)propyl)amino)ethyl)amino)-3-oxopropyl)amino)-3-oxopropylamine dihydrochloride **(7)** (2.2 mg, 4.54 µmol) in DMF (0.2 mL), DIPEA (1.98 µL, 11.40 µmol) was added and then BODIPY 630/650-X-SE (1.5 mg, 2.27 µmol) dissolved in DMF (0.8 mL) was added. The mixture was stirred with the exclusion of light for 3 hours. The solvent was removed under reduced pressure and the crude material was purified by semi-preparative HPLC (Method B) to give the title compound as a blue solid (2.0 mg, 91%). Analytical RP-HPLC t_R = 19.68 min, purity = 98% HRMS (ESI-TOF) *m/z* calc. for $C_{50}H_{57}BF_2N_7O_7S$ $[M+H]^+$; 948.4098 found; 948.4096 and 970.3929 [M+Na].

cDNA Construct.

The β2-adrenoceptor cDNA sequence (obtained from Missouri S&T cDNA Resource Centre; www.cdna.org) was PCR amplified to generate a β2-adrenoceptor sequence that was in frame with the BamH1 restriction site of sig-NLuc (Stoddart et al., 2015) and sig-SNAP

(Gherbi et al., 2015) and changed the start codon (Met) of the β2-adrenoceptor sequence to Leu. The primers used were: forward 5'-CCGCCGGATCCCTGGGGCAACCCGGGAACG-3' and reverse 5'-GGCGGGAATTCTTACAGCAGTGAGTCATTTG-3'. The PCR product was then ligated in frame into pcDNA3.1(+) containing sig.SNAP[3] or sig-NLuc[1] using BamHI and EcoRI restriction enzymes. This created the plasmids sig-SNAP-ADRB2-pcDNA3.1(+) and sig-NLuc-ADRB2-pcDNA3.1(+).

The pSIN-SNAP-ADRB2 construct was generated on the basis of pSIN-eGFP-BSD lentiviral vector (Dixon et al., 2011) as following: first, the pSIN-eGFP-BSD plasmid was digested with SpeI and EcoRI restriction enzymes in order to remove eGFP sequence and to produce the pSIN/BSD backbone; second, SNAP-ADRB2 fragment was PCR amplified with 5'-CTTAAACTAGTTACCGCCACCATGCGGCTCTGC-3' (forward) and 5'-TCTGCAGAATTCttacagcagtgagtcatttg-3' (reverse) primers using sig-SNAP-ADRB2-pcDNA3.1(+) as a template. The resulting PCR product was digested with SpeI and EcoRI restriction endonucleases and ligated into eGFP-free pSIN/BSD backbone. To make the pSIN-NLuc-ADRB2 construct, a NheI-EcoRI fragment containing the NanoLuc-ADRB2 fusion sequence was isolated from sig-NLuc-ADRB2-pcDNA3.1(+) and used to replace SpeI-EcoRI SNAP-ADRB2 sequence in pSIN-SNAP-ADRB2.

Cancer cell model.

This study used a highly metastatic variant of the female MDA-MB-231 triple-negative human breast cell line (MDA-MB-231^{HM}; a kind gift from Dr. Zhou Ou, Fudan University Shanghai Cancer Center, China) (Chang et al., 2008). Cell line identity was confirmed by short tandem repeat analysis. The cell line was stably transfected with the lentiviral vector pSIN-NLuc-ADRB2 encoding Nanoluc-β_2-adrenoreceptor (Nluc-β_2AR), using Fugene^{HD} reagent, following the manufacturer's protocol, using a 3:1 reagent:DNA ratio. Transfected cells were selected using 20 µg/mL blastocidin (Sigma). 10 µg/mL blastocidin was used for cell maintenance. Cells were cultured in Dulbecco's Modified Eagle Medium (DMEM) containing 2mM Glutamax (Gilbco) supplemented with 10% foetal bovine serum. For in vivo use, blastocidin was removed from the growth medium for several passages.

Confocal microscopy.

Confocal microscopy was performed using a Leica TCS SP8 inverted scanning microscope with a Zeiss 40x 1.3NA oil immersion HCPL APO CS2 objective lens. Untransfected MDA-MB-231^{HM} cells or MDA-MB-231^{HM} cells expressing NanoLuc-tagged β2-adrenoceptors were seeded in eight-well chambered coverslip slides (µSlide; Ibidi, Martinsried, Germany). Prior to imaging, media was replaced with Hank's buffered salt solution (HBSS) (Gibco, Thermo Fisher) pH 7.2-7.4, at 37°C. Cells were incubated in HBSS for 10 min with the nuclear stain

Hoechst 33342 (2 μg/mL), and then washed twice with HBSS. Cells were then treated with 50nM propranolol-(β-Ala-β-Ala)-X-BODIPY630/650, in the presence or absence of unlabelled propranolol (10μM), and incubated for 30 min at 37°C (without CO_2). Cells were washed with HBSS to remove unbound ligand before imaging. Nuclear labelling was detected using a 405nm-Argon laser line (415-470nm bandpass), and fluorescent ligand labelling was detected using a 633nm HeNe laser line and a 640-700 bandpass filter. Images were analysed using ImageJ 1.51 (Fiji, USA) software.

Widefield bioluminescence microscopy.

Bioluminescence imaging was performed using an Olympus LV200 Wide field inverted microscope, equipped with a 60x/1.42NA oil immersion objective lens. MDA-MB-231^{HM} cells transfected with NanoLuc-tagged human β_2-adrenoceptors were seeded into a 35mm MatTek dish containing a high tolerance 1.5μm coverslip. Before imaging, media was removed and cells were incubated with 2mL HBSS containing 400nM furimazine substrate (Promega) at 37°C, for 15 min. Background luminescence images were taken by capturing sequentially luminescence in the following channels: (1) open channel (20 sec exposure time); (2) DAPI channel (20 sec exposure time; 420nm longpass filter) and (3) Cy5 channel (4 min exposure time; 600/50nm bandpass filter). Cells were then incubated for 30 min with 50nM propranolol-(β-Ala-β-Ala)-X-BODIPY630/650, in the presence or absence of 10 μM ICI 118551, before images were acquired using the same acquisition sequence. BRET ratio measurements were performed using ImageJ 1.51 (National Institutes of Health, USA) and the time-series analyser V3 plugin.

***In vitro* NanoBRET assays.**

Saturation, competition and kinetics NanoBRET binding assays were performed on MDA-MB-231^{HM} cells stably expressing NanoLuc-tagged β2-adrenoceptors as described previously (Stoddart et al., 2015). Briefly, cells were seeded 24h before assay in white Perkin Elmer 96-well Isoplates. For experiments performed under equilibrium conditions, growth media was replaced with 100μL HBSS. Fluorescent and non-fluorescent ligands were added simultaneously and the 96-well plate was incubated for 1h at 37 °C (no CO_2). 10μL NanoLuc substrate furimazine (Promega) was then added to give a final concentration of 10μM and the plate was incubated for a further 5 minutes at 37 °C. For all experiments, the luminescence was measured using a CLARIOstar plate reader (BMG Labtech) with filtered light emission collected at 685nm/100nm bandpass (acceptor) and 460nm/80nm bandpass (donor) at room temperature. The raw BRET ratio was calculated by dividing the 685nm emission by the 460nm emission. The same experiments were also performed using the IVIS Lumina II whole-animal imaging system (Caliper Life Sciences, Perkin Elmer) using

both an open channel (donor; 1 sec exposure time) and the CY5.5 channel (acceptor; 660/20nm bandpass; 30 sec exposure time).

For kinetics binding assays, growth media from cells was replaced with 50µL HBSS containing 10µM furimazine substrate, and incubated for 15 min, at 37°C (without CO_2). 50µL ligands, previously prepared in HBSS, were then added to wells and luminescence measurements were made every minute (for 60 min) with the above emission settings on both the CLARIOstar plate reader and the IVIS Lumina II system as described above.

Breast cancer *in vivo* model.

$5x10^5$ (in 20µl PBS) MDA-MB-231^{HM} triple negative human breast cancer cells stably expressing Nanoluc-β_2-adrenoceptors were injected (Hamilton syringe with 26.5G needle) into the fourth left mammary fat pad of female BALB/c nu/nu immune-compromised mice (7-week-old) (University of Adelaide, Australia). Mice were housed under PC2 barrier conditions on a 12 h dark/light cycle and monitored daily. Primary tumours were measured by caliper and volume (mm^3) was calculated by the formula: (length x width^2)/2. Mice were maintained under 2-3% isoflurane anaesthesia during injections and imaging. All *in vivo* procedures were carried out at Monash Institute of Pharmaceutical Sciences according to protocols approved (MIPS.2012.11) by the Monash University Animal Ethics Committee and according to the NHMRC (Australia) guidelines.

Bioluminescence imaging to monitor tumour and metastasis development.

The growth of solid tumours and the extent of metastasis were monitored by whole-animal bioluminescence imaging. The luminescence from the NanoLuc-β2-adrenoceptors on MDA-MB-231^{HM} cells was monitored using an IVIS Lumina II camera system equipped with a heated stage (37°C). On the day of imaging, mice were anaesthetised with 2-3% isoflurane and then injected via the tail vein (i.v.) with 100µl Nano-Glo® luciferase substrate (furimazine, diluted in PBS; *circa* 0.37 mg/kg). Whole-animal images were taken 5 min after substrate injection (open channel; 30 sec exposure time). Immediately afterwards, images of metastasis localised in the thorax region were also taken. Images of the thorax were acquired 10 min after furimazine injection, using luminescence imaging (open channel; 2 min exposure time).

***In vivo* NanoBRET.**

i) Monitoring fluorescent ligand-receptor association in the primary tumour

In preliminary experiments to monitor ligand-receptor association *in vivo*, mice were firstly injected with 0.1 mg/kg Propranolol-(β-Ala-β-Ala)-X-630/650 (in 50µl of PBS) directly into the primary tumour (intratumoral; i.t). Immediately after fluorescent ligand injection, mice were

injected with 100μL 1:20 dilution furimazine substrate via the tail vein (i.v., 100μL diluted in PBS; *circa* 0.37 mg/kg) and imaged using the IVIS lumina II camera system. Mice were kept under 2-3% isoflurane anaesthesia during injections and imaging. Sequential luminescence (open channel, 30 sec exposure time) and fluorescence (Cy5.5. channel using 660/20nm bandpass filter; 5 min exposure time) images were taken every 6 min for a total time of 49 min. Images were acquired from 1 min after fluorescent ligand injection.

ii) Monitoring fluorescent ligand-receptor dissociation in the primary tumour

To monitor fluorescent ligand dissociation over-time in the primary tumour region, mice were administered with one of three different doses of Propranolol-(β-Ala-β-Ala)-X-630/650 (0.01, 0.03 or 0.1 mg/kg). At 1, 24, 48 and 72h after fluorescent ligand injection, mice were injected with furimazine substrate (i.v., 100μL in PBS, *circa* 0.37 mg/kg) and imaged 5 min later using the IVIS lumina II camera system. Sequential luminescence (open channel, 30 sec exposure time) and fluorescence (Cy5.5 channel, 5 min exposure times) images were acquired at 1h, 24h, 48h and 72h after fluorescent ligand injection (i.t.). All mice were also imaged on the day before fluorescent ligand injection, 5 min after 100μL i.v. injection with furimazine substrate (1:20 dilution in PBS) to determine luminescence (and BRET) baseline.

iii) Monitoring ligand-receptor engagement of unlabelled drugs administered locally in the primary tumour (i.t.) or via intravenous injection (i.v.)

To monitor specific ligand-receptor engagement of the unlabelled β_2-selective antagonist, ICI 118551, administered locally in the primary tumour (i.t.), mice were divided into two groups: Group 1 received an intra-tumour (i.t. 0.1 mg/kg; 50μL in PBS) injection of the fluorescent ligand alone and Group 2 received 0.3 mg/kg (i.t.; 50μL in PBS) ICI 118551 45 min prior to injection of 0.1 mg/kg Propranolol-BY630 (i.t.). 1h after the fluorescent ligand injection luminescence/fluorescence images were captured on the IVIS lumina II instrument (NanoLuc donor open channel, 30 sec exposure time; BRET acceptor Cy5.5 channel, 660nm/20nm band pass, 5 min exposure time), 5 min after furimazine injection (100μL in PBS; circa 0.37 mg/kg). Mice were imaged on the previous day 5 min after an equivalent furimazine i.v. injection to determine luminescence (and BRET) baseline. After 10 days, when fluorescent ligand was no longer detected by imaging, the treatment schedule was reversed. Group 1 mice were injected with 0.3 mg/kg ICI 118551 plus 0.1 mg/kg Propranolol-BY630/650, while Group 2 animals were injected with fluorescent ligand alone.

To investigate drug-receptor engagement in the primary tumour region following parenteral administration (i.v.) of the selective β_2-selective antagonist ICI 118551 (100μl in PBS; 10 mg/kg) a similar cross over experimental design was followed with 0.1 mg/kg propranolol-(β-Ala-β-Ala)-X-BODIPY630/650 injected directly into the primary tumour 45 min after i.v.

administration of ICI 118551. Mice were also imaged on the previous day, 5 min after i.v. injection of furimazine to determine luminescence (and BRET) baseline. Images were acquired as described above using the IVIS system, 5 min after furimazine substrate (100μL in PBS; 0.37 mg/kg) injection, using the same filter settings and exposure times as described above for the donor and acceptor readings.

Drug-receptor engagement in the primary tumour region was also investigated for a lower dose of ICI 118551 administered i.v. (1 mg/kg), as well as for an unlabelled β_1-selective antagonist, CGP20712A administered i.v. (10 mg/kg; 100μL in PBS). In these experiments, mice were administered with fluorescent ligand (0.1 mg/kg i.t.; 50μL in PBS). 45 min prior to fluorescent ligand injection, mice were administered (i.v.) with either PBS (100μL), CGP201712A (100μL; 10 mg/kg in PBS), or ICI 118551 (100μL; 1 mg/kg in PBS). Images were acquired 1h after fluorescent ligand injection, as described above. All mice were also imaged on the previous day, 5 min after i.v. injection with furimazine substrate (1:20 dilution, *circa* 0.37 mg/kg) to determine luminescence (and BRET) baseline. BRET ratios were calculated after dividing acceptor/donor emissions (photons/sec), determined using regions of interest (ROIs) drawn over the tumour site.

Data analysis.

***In vitro* pharmacological characterisation.**

For analysis of saturation binding data, raw BRET ratios obtained from each individual experiment were fitted using a non-linear regression equation shown below, using GraphPad Prism 7. Total and non-specific binding curves were fitted simultaneously using the following equation:

$$BRET\ Ratio = \frac{B_{max} \times [B]}{[B] + K_D} + \left((M \times [B]) + C\right)$$

where B_{max} is the maximal binding, [B] is the concentration of fluorescent ligand, K_D is the equilibrium dissociation constant, M is the slope of the non-specific binding component and C is the intercept with the Y-axis.

Competition binding curves were fitted to the following equation using Prism 7:

$$\%\ uninhibited\ binding = \frac{(100-NS)}{([A]/IC_{50})+1} + NS$$

where [A] is the concentration of unlabelled, IC_{50} is the molar concentration of the unlabelled ligand required to inhibit 50% of the specific binding of 50nM propranolol-(β-Ala-β-Ala)-X-BODIPY630/650 and NS represents non-specific binding.

The Cheng Prusoff equation was then used to convert IC_{50} values to absolute K_i values:

$$K_i = \frac{IC_{50}}{1+\frac{[L]}{K_D}}$$

where [L] is the concentration of labelled ligand and K_D is the dissociation constant of the fluorescent ligand obtained from saturation binding assays. pK_i values were then calculated as –log K_i.

Data obtained for fluorescent ligand binding kinetics, using more than one concentration, were globally fitted to an association kinetics model. The kinetic rate constants: k_{off} (dissociation rate constant of the ligand; min^{-1}) and k_{on} (association rate constant; M^{-1} min^{-1}) were calculated from the following equation:

$$k_{on} = \frac{k_{obs} - k_{off}}{[L]}$$

where [L] is the fluorescent ligand concentration and k_{obs} is calculated from global fitting of the data to the following monoexponential association function:

$$Y = Y_{max}(1 - e^{-k_{obs}t})$$

where Y is the specific binding at time t, $Ymax$ corresponds to the level of specific binding at infinite time and k_{obs} is the rate constant for the observed rate of association.

***In vivo* data analysis.**

In vivo luminescence or fluorescence total flux (photons/sec) measurements were obtained using ROIs positioned on the primary tumour or thorax region, for primary tumour or metastasis measurements, respectively. Raw BRET ratios were calculated as:

$$BRET\ ratio = \frac{Acceptor\ Luminescence\ (CY5.5)}{Donor\ Luminescence\ (open\ channel)}$$

where acceptor luminescence (Cy5.5 channel) is measured as total flux (photons/sec) acquired using the Cy5.5 emission channel (660nm/20nm bandpass), and luminescence (open channel) is measured as total flux (photons/sec) acquired without using emission filters.

Statistical analysis.

For *in vitro* and *in vivo* studies, one-way or two-way ANOVA analysis with Tukey's or Dunnett's multiple comparison tests were used.

Supplemental References.

Chang, X.Z., Li, D.Q., Hou, Y.F., Wu, J., Lu, J.S., Di, D.H., Jin, W., Ou, Z.L., Shen, Z.Z., Shao, Z.M. (2008) Identification of the functional role of AF1Q in the progression of breast cancer. Breast Cancer Res. Treat. 111, 65–78.

Dixon, J. E. Dick, E., Rajamohan, D., Shakesheff, K.M., and Denning, C. (2011) Directed Differentiation of Human Embryonic Stem Cells to Interrogate the Cardiac Gene Regulatory Network. Molecular Therapy 19, 1695-1703.

Gherbi, K., May, L.T., Baker, J.G., Briddon, S.J., and Hill, S.J. (2015) Negative cooperativity across β_1-adrenoceptor homodimers provides insights into the nature of the secondary low affinity “CGP 12177” β_1-adrenoceptor binding conformation. *FASEB J.* 29, 2859-71.

Soave, M., Stoddart. L.A., Brown. A., Woolard. J., and Hill. S.J. (2016) Use of a novel proximity assay (BRET) to investigate the ligand binding characteristics of three fluorescent ligands to the human β1-adrenocpetor expressed in HEK-293 cells. Pharmacol. Res. Persp. 4: e00250.

Stoddart, L.A., Johnstone, E.K.M., Wheal, A.J., Goulding, J., Robers, M.B., Machleidt, T., Wood, K.V., Hill, S.J., and Pfleger KDG. (2015) Application of BRET to monitor ligand binding to GPCRs. Nature Methods, 12, 661–663.
